# Crystal structure of the ferredoxin reductase component of carbazole 1,9a-dioxygenase from *Janthinobacterium* sp. J3

**DOI:** 10.1107/S2059798321005040

**Published:** 2021-06-18

**Authors:** Yuji Ashikawa, Zui Fujimoto, Kengo Inoue, Hisakazu Yamane, Hideaki Nojiri

**Affiliations:** a Agro-Biotechnology Research Center, Graduate School of Agricultural and Life Sciences, The University of Tokyo, 1-1-1 Yayoi, Bunkyo-ku, Tokyo 113-8657, Japan; b Advanced Analysis Center, National Agriculture and Food Research Organization, 2-1-2 Kannondai, Tsukuba, Ibaraki 305-8518, Japan; c Department of Biochemistry and Applied Biosciences, Faculty of Agriculture, University of Miyazaki, 1-1 Gakuenkibanadai-nishi, Miyazaki 889-2192, Japan; d Agricultural Bioinformatics Research Unit, Graduate School of Agricultural and Life Science, The University of Tokyo, 1-1-1 Yayoi, Bunkyo-ku, Tokyo 113-8657, Japan; e Collaborative Research Institute for Innovative Microbiology, The University of Tokyo, 1-1-1 Yayoi, Bunkyo-ku, Tokyo 113-8657, Japan

**Keywords:** Rieske nonheme iron oxygenase, NAD(P)H:ferredoxin oxidoreductase, ferredoxin, *Janthinobacterium* sp. J3, carbazole 1,9a-dioxygenase, CARDO, electron transfer

## Abstract

Carbazole 1,9a-dioxygenase (CARDO), which consists of an oxygenase component and the electron-transport components ferredoxin (CARDO-F) and ferredoxin reductase (CARDO-R), is a Rieske nonheme iron oxygenase (RO). ROs are classified into five subclasses (IA, IB, IIA, IIB and III) based on their number of constituents and the nature of their redox centres. In this study, two types of crystal structure (type I and type II) were resolved of the class III CARDO-R from *Janthinobacterium* sp. J3 (CARDO-R_J3_). Superimposition of the type I and type II structures revealed the absence of flavin adenine dinucleotide (FAD) in the type II structure along with significant conformational changes to the FAD-binding domain and the C-terminus, including movements to fill the space in which FAD had been located. Docking simulation of NADH into the FAD-bound form of CARDO-R_J3_ suggested that shifts of the residues at the C-terminus caused the nicotinamide moiety to approach the N5 atom of FAD, which might facilitate electron transfer between the redox centres. Differences in domain arrangement were found compared with RO reductases from the ferredoxin–NADP reductase family, suggesting that these differences correspond to differences in the structures of their redox partners ferredoxin and terminal oxygenase. The results of docking simulations with the redox partner class III CARDO-F from *Pseudomonas resinovorans* CA10 suggested that complex formation suitable for efficient electron transfer is stabilized by electrostatic attraction and complementary shapes of the interacting regions.

## Abstract 2

The crystal structure of the ferredoxin reductase component of carbazole 1,9a-dioxygenase from *Janthinobacterium* sp. J3 was determined at 2.40 Å resolution.

## 1. Introduction   

Rieske nonheme iron oxygenases (ROs) are the initial catalysts in the degradation pathways of numerous environmentally hazardous aromatic compounds and components of crude oil (Mason & Cammack, 1992 ▸; Nojiri & Omori, 2002 ▸; Nojiri, 2012 ▸). Few enzymes catalyse the introduction of O atoms into stable aromatic hydrocarbon substrates. ROs, one group of such enzymes, have been suggested to use a novel oxygen activation and addition mechanism. With very few exceptions, ROs catalyse the incorporation of both O atoms from molecular dioxygen onto tandemly linked C atoms of an aromatic ring, forming two hydroxyl groups in the *cis* configuration. ROs generally consist of two or three discrete components that form an electron-transfer chain from NAD(P)H via flavin and [2Fe–2S] redox centres to the site of dioxygen activation (Bugg & Ramaswamy, 2008 ▸). ROs have been classified into five groups, IA, IB, IIA, IIB and III, based on the number of constituents and the nature of their redox centres (Batie *et al.*, 1991 ▸; Ferraro *et al.*, 2005 ▸). Class I ROs consist of reductase and oxygenase components, with their reductase components containing both flavin [flavin mononucleotide (FMN) in class IA and flavin adenine dinucleotide (FAD) in class IB] and a chloroplast-type [2Fe–2S] cluster. Both class II and class III ROs contain a ferredoxin component in addition to reductase and oxygenase components. Class II is further divided into classes IIA and IIB, which use putidaredoxin-type and Rieske-type ferredoxins, respectively. Class II reductases contain FAD as the only cofactor, whereas class III reductases contain FAD and a chloroplast-type [2Fe–2S] cluster. Although there is variation in the redox-transfer machineries of both the reductase and the ferredoxin, these components transfer electrons from NAD(P)H to oxygenase for dioxygen activation.

Carbazole 1,9a-dioxygenase (CARDO), which has been isolated from various carbazole-degrading bacteria, is an RO that catalyses the initial dioxygenation reaction in the carbazole-degradation pathway (Nojiri & Omori, 2007 ▸; Inoue *et al.*, 2004 ▸, 2005 ▸; Vejarano *et al.*, 2018 ▸, 2019 ▸). All reported CARDOs consist of three components: the terminal oxygenase CARDO-O, the ferredoxin CARDO-F and the ferredoxin reductase CARDO-R (Supplementary Fig. S1). CARDO-O is a homotrimeric enzyme that contains one Rieske-type [2Fe–2S] cluster and one active-site iron (Fe^2+^) in a single subunit. The electron-transport proteins of CARDO, which mediate electron transport from NAD(P)H to CARDO-O, comprise CARDO-F, which contains one Rieske-type [2Fe–2S] cluster, and CARDO-R, which contains one FAD and one plant-type [2Fe–2S] cluster. The CARDOs from *Pseudomonas resinovorans* CA10, *Janthinobacterium* sp. J3, *Novosphingobium* sp. KA1 and *Nocardioides aromaticivorans* IC177 are grouped into classes III, III, IIA and IIB, respectively (Sato *et al.*, 1997 ▸; Inoue *et al.*, 2004 ▸, 2006 ▸; Urata *et al.*, 2006 ▸), indicating that their CARDOs include diverse types of electron-transfer components (CARDO-F and CARDO-R). Although the structures of several RO components are known (Ferraro *et al.*, 2005 ▸; Senda *et al.*, 2007 ▸; Lin *et al.*, 2012 ▸), the precise nature of their electron-transfer mechanism remains unclear. Therefore, CARDOs provide an excellent model system for studying the structure–function relationships of RO components and the mechanism of electron transfer.

To date, crystal structures of the CARDO-F from *P. resinovorans* CA10 (CARDO-F_CA10_; Nam *et al.*, 2005 ▸), CARDO-O from *Janthinobacterium* sp. J3 (CARDO-O_J3_; Nojiri *et al.*, 2005 ▸) and the electron-transfer complex between CARDO-O_J3_ and CARDO-F_CA10_ (Ashikawa *et al.*, 2005 ▸, 2006 ▸) have been identified. The structures of the class IIB CARDO-O and CARDO-F from *N. aromaticivorans* IC177 (CARDO-O_177_ and CARDO-F_IC177_) have also been determined (Inoue *et al.*, 2009 ▸). However, no CARDO-R structures have yet been successfully determined. Considering that no class III RO reductase containing a plant-type [2Fe–2S] cluster and FAD has been structurally elucidated, structural and functional studies of a class III CARDO-R are needed to clarify the diversity of electron-transfer functions.

RO reductases belong to two distinct families: the ferredoxin–NADP reductase (FNR) family and the glutathione reductase (GR) family. The structures of phthalate dioxygenase reductase (PDO-R; class IA) from *Pseudomonas cepacia* PHK (Correll *et al.*, 1992 ▸) and benzoate dioxygenase reductase (BZDO-R; class IB) from *Acinetobacter baylyi* ADP1 (Karlsson *et al.*, 2002 ▸), both of which belong to the FNR family, have been determined. Both of these reductases are composed of a domain containing a plant-type [2Fe–2S] cluster, an FAD-binding domain and an NADH-binding domain. In addition, structures of the reductase components of biphenyl dioxygenase from *Pseudomonas* sp. KKS102 and of toluene dioxygenase from *P. putida* F1, which belong to the GR family, have been determined as examples of class IIB RO reductases (Senda *et al.*, 2000 ▸; Lin *et al.*, 2012 ▸). These reductases consist of three domains: an NADH-binding domain, an FAD-binding domain and a C-terminal domain corresponding to the interface domain in the GR family. In CARDO systems, the class III CARDO-R_CA10_ and CARDO-R_J3_ belong to the FNR family, while the class IIB CARDO-R_IC177_ and the class IIA CARDO-R from *Novosphingobium* sp. KA1 (CARDO-R_KA1_) are classified into the GR family. Here, we report the crystal structure of CARDO-R_J3_, providing the first description of the crystal structure of a class III RO reductase. This report makes the complete series of structures of all CARDO system components available for the first time. Using this newly determined structure, we investigated the binding of NADH to CARDO-R_J3_ and the formation of the electron-transfer complex using docking simulations. Furthermore, we assessed the electron-transfer interaction between CARDO-R and its cognate CARDO-F using molecular-docking simulations. These results strengthen our understanding of how the interactions between discrete components can affect complex formation and electron transfer in the RO system.

## 2. Materials and methods   

### 2.1. Purification and crystallization   

The ferredoxin reductase component from* Janthinobacterium* sp. J3 (CARDO-R_J3_) was expressed in *Escherichia coli* and purified as described previously (Ashikawa *et al.*, 2007 ▸). The crystallization conditions used to obtain the two types of crystals have been described previously (Ashikawa *et al.*, 2007 ▸). The selenomethionine (SeMet) substituent of CARDO-R_J3_ was expressed in the methionine-auxotrophic *E. coli* strain B834 (DE3) (Novagen). *E. coli* strain B834 (DE3) transformed with pEJ3NAd was grown in medium containing SeMet (Doublié & Carter, 1992 ▸) and was purified and crystallized using the same methods as used for native CARDO-R_J3_.

### 2.2. Data collection   

X-ray diffraction data were collected from the CARDO-R_J3_ crystals at 100 K at a wavelength of 1.0 Å on beamline NW12A at the Photon Factory Advanced Ring, High Energy Accelerator Research Organization. Diffraction data were collected from the native crystals and processed with *CrystalClear* (Rigaku, Japan). The data-collection and processing statistics are given in Table 1 ▸.

### 2.3. Single-wavelength and multi-wavelength anomalous diffraction (SAD and MAD) phasing   

The structure of the type I CARDO-R_J3_ crystal (Ashikawa *et al.*, 2007 ▸) was determined by single-wavelength anomalous diffraction (SAD) and multi-wavelength anomalous diffraction (MAD) experiments using the anomalous scattering of selenium in the SeMet-substituted crystal (peak 0.97945 Å) and of iron in the native crystal (peak 1.73974 Å, edge 1.73993 Å and remote 1.69243 Å), respectively (Table 1 ▸). The collected data were processed and scaled using *HKL*-2000 (Otwinowski & Minor, 1997 ▸). Both types of phase calculation were performed using *SOLVE*/*RESOLVE* (Terwilliger & Berendzen, 1999 ▸; Terwilliger, 2000 ▸) and *SHARP*/*autoSHARP* (Vonrhein *et al.*, 2007 ▸). The positions of 18 strong selenium peaks (seven Se atoms per molecule) and three large iron peaks (two Fe atoms per molecule) were determined (figures of merit of 0.35 and 0.47, respectively). The initial phase obtained from the Fe-MAD experiment was not sufficient to construct the model structure, and the initial structure was determined by improving the phase using the Se-SAD data.

### 2.4. Structure refinement   

Building of the initial model of the type I crystal from the electron-density map was carried out with *QUANTA* (Accelrys, San Diego, California, USA) and *Coot* (Emsley *et al.*, 2010 ▸). Refinement was conducted using *REFMAC*5 in *CCP*4 (Winn *et al.*, 2011 ▸) and *CNS* 1.1 (Brünger *et al.*, 1998 ▸) through the gradual addition of water molecules. The type II structure was determined by molecular replacement with *Phaser* (McCoy *et al.*, 2007 ▸) using the type I structure, and model building and refinement were performed using the programs listed above. The stereochemistry of the model was analysed using *PROCHECK* (Laskowski *et al.*, 1993 ▸), *WHATCHECK* (Hooft *et al.*, 1996 ▸), *RAMPAGE* (Lovell *et al.*, 2003 ▸) and *MolProbity* (Chen *et al.*, 2010 ▸). The refinement statistics are listed in Table 1 ▸.

### 2.5. NAD(P)H docking simulation   

CARDO-R_J3_ was superimposed with pea FNR in complex with NADPH (PDB entry 1qfz; Deng *et al.*, 1999 ▸) using *Coot* to build an initial model of NADH and NADPH binding. Some changes in the predicted positions of the NAD(P)H–CARDO-R_J3_ complexes were performed considering protein–ligand interactions. After building the final models, the position and geometry of NAD(P)H in the CARDO-R_J3_ structure were minimized using *Phenix* (Liebschner *et al.*, 2019 ▸).

### 2.6. Docking simulations between CARDO-R_J3_ and CARDO-F_CA10_   

Docking simulation between CARDO-R_J3_ and CARDO-F_CA10_ (PDB entry 1vck; Nam *et al.*, 2005 ▸) was performed using the *ClusPro* web server (http://nrc.bu.edu/cluster; Comeau *et al.*, 2004*a* ▸,*b* ▸). Rigid-body docking was performed with a scoring function based on shape complementarity, electrostatic potential and desolvation terms. Predictions for fitting the ligand protein (CARDO-F_CA10_) to the receptor protein (CARDO-R_J3_) were then filtered using residues ligated to the [2Fe–2S] cluster of CARDO-F_CA10_ and a 6 Å distance cutoff, and were clustered (9 Å clustering radius) and ranked using the automated *ClusPro* web server. The ligand protein with the greatest number of neighbours was the cluster centre, and this number was minimized using the *CHARMM* algorithm in the presence of the receptor protein.

## 3. Results and discussion   

### 3.1. Quality of structures   

**Figure 1:**
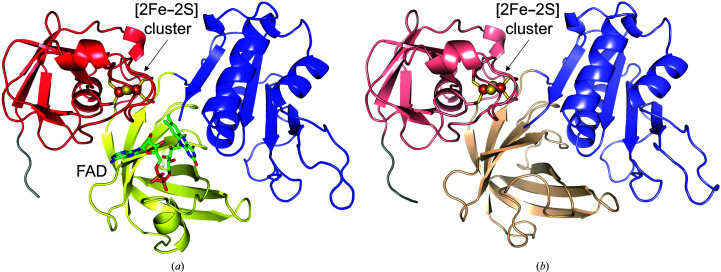
Structures of the type I and type II crystals of CARDO-R_J3_. (*a*) Type I overall structure shown in cartoon presentation. The Fd domain is coloured red, the FAD-binding domain is in yellow and the NADH-binding domain is in blue. Bound FAD (green sticks) and the [2Fe–2S] cluster (brown and yellow spheres) are also shown. (*b*) Type II overall structure shown in the same manner as the type I structure (Fd domain, salmon; FAD-binding domain, wheat; NADH-binding domain, slate). The [2Fe–2S] cluster is shown as brown and yellow spheres. His tags at the N-termini of molecules are shown in grey. All molecular graphics in this paper were prepared using *PyMOL* (DeLano, 2002 ▸).

In a previous study, we obtained two types of CARDO-R_J3_ crystals (type I and type II; Ashikawa *et al.*, 2007 ▸). Refinement statistics for the two crystal structures of CARDO-R_J3_ are summarized in Table 1 ▸. The type I crystals were resolved using SAD (Se atoms) and MAD (Fe atoms) experiments. The type I structure was refined at 2.6 Å resolution with a crystallo­graphic *R* factor of 0.249 (*R*_free_ = 0.292; Fig. 1 ▸ *a*). The type I structure contained three CARDO-R_J3_ molecules per asymmetric unit (Supplementary Fig. S2*a* ); one residue (Leu289 in chain *C*) fell into the outlier region of the Ramachandran plot created using *RAMPAGE* (Lovell *et al.*, 2003 ▸). Superposition of the three molecules yielded a root-mean-square deviation (r.m.s.d.) value of 0.79 Å for 313 C^α^ atoms. The type II crystal structure was determined by the molecular-replacement method using the type I structure and was refined at 2.4 Å resolution with a crystallographic *R* factor of 0.227 (*R*_free_ = 0.281; Fig. 1 ▸ *b*). The asymmetric unit contained three CARDO-R_J3_ polypeptide chains, as in the type I structure. One residue, Leu289 in chain *B*, fell outside the allowed regions of the Ramachandran plot (Lovell *et al.*, 2003 ▸). Superposition of the three molecules yielded an r.m.s.d. value of 0.88 Å for 301 C^α^ atoms. The three molecules in the asymmetric unit of each structure were connected by a noncrystallographic threefold axis, although CARDO-R_J3_ was a monomer in solution. In both structures, electron density for one Ni^2+^ ion, which was deduced by analyses using the anomalous Fe-MAD map and *CheckMyMetal* (Zheng *et al.*, 2014 ▸), was observed on this axis and coordinated by two histidine residues of the N-terminal His tag from each of the three molecules (Supplementary Fig. S2*b* ). Some disordered regions were present in all molecules in both structures. The type I structure contained the [2Fe-2S] cluster and FAD, while the type II structure lacked FAD (the apo form), which is essential for the physiological function of CARDO-R_J3_. In addition, in both structures we observed chloride ions and iodine ions, which were contained in the crystallization buffer and possess larger electron densities compared with water molecules (Supplementary Fig. S2*c* ). Both structures did not contain NAD(P)H.

### 3.2. Overall structure of CARDO-R_J3_   

**Figure 2:**
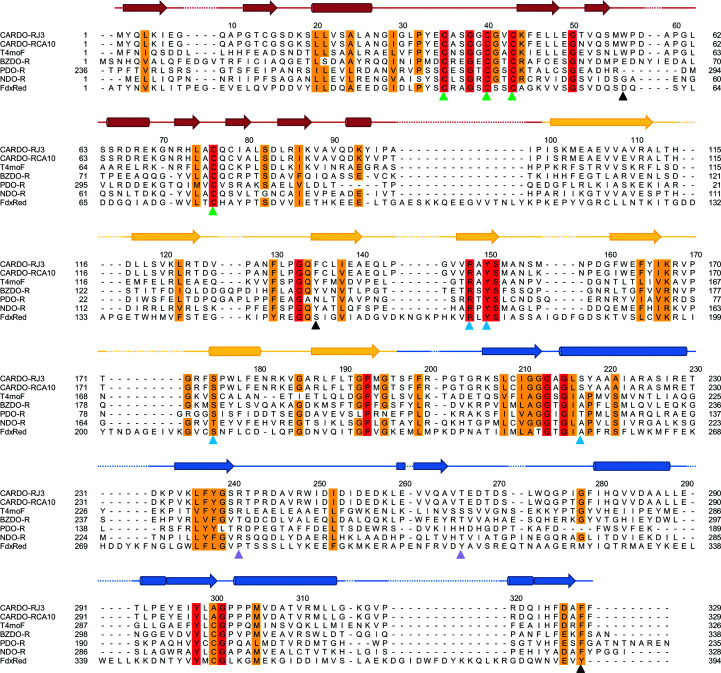
Structure-based alignment of the amino-acid sequence of *Janthinobacterium* sp. J3 ferredoxin reductase (CARDO-RJ3; UniProt entry Q84II0) with related sequences. Structural alignment with carbazole 1,9a-dioxygenase ferredoxin reductase from *Pseudomonas resinovorans* CA10 (CARDO-RCA10; UniProt entry Q84GI4), toluene 4-monooxygenase NADH oxidoreductase from *P. mendocina* (T4moF; PDB entry 4wqm), benzoate dioxygenase reductase from *Acinetobacter baylyi* ADP1 (BZDO-R; PDB entry 1krh), phthalate dioxygenase reductase from *P. cepacia* (PDO-R; PDB entry 2pia), naphthalene dioxygenase reductase from *P. putida* (NDO-R; UniProt entry Q52126) and the FNR–ferredoxin complex from maize leaf (FdxRed; PDB entry 1gaq) was performed using *PROMALS*3*D* (Pei *et al.*, 2008 ▸). Secondary-structure assignments and the colour scheme are based on the CARDO-R_J3_ structure (Fig. 1 ▸ *a*). The red and orange columns indicate completely conserved and highly homologous amino-acid residues, respectively. The C-terminal ferredoxin-like domain (beginning at Thr236) of PDO-R aligns with the N-terminal ferredoxin-like domains (Fd domains) of the other reductase sequences. The solid green arrows show the four cysteine ligands of the [2Fe–2S] cluster. Light blue solid arrows indicate residues with direct side-chain hydrogen bonds to FAD, and the solid black arrows indicate aromatic residues that exhibit stacking interactions with FAD. The purple arrows show the amino-acid residues that form hydrogen bonds to NADH via their side chains in the docking simulation.

CARDO-R_J3_ is a 329-residue monomeric enzyme that belongs to the FNR superfamily. It consists of three domains: an N-terminal ferredoxin (Fd) domain (residues 1–99), an FAD-binding domain (residues 100–196) and an NADH-binding domain (residues 197–329) (red, yellow and blue regions, respectively, in Fig. 2 ▸). The N-terminal Fd domain contains the plant-type [2Fe–2S] cluster. The FAD-binding domain is in a central position in the molecule and interacts with the Fd domain on one side and the NADH-binding domain on the other side; the Fd and NADH-binding domains have little direct interaction.

**Figure 3:**
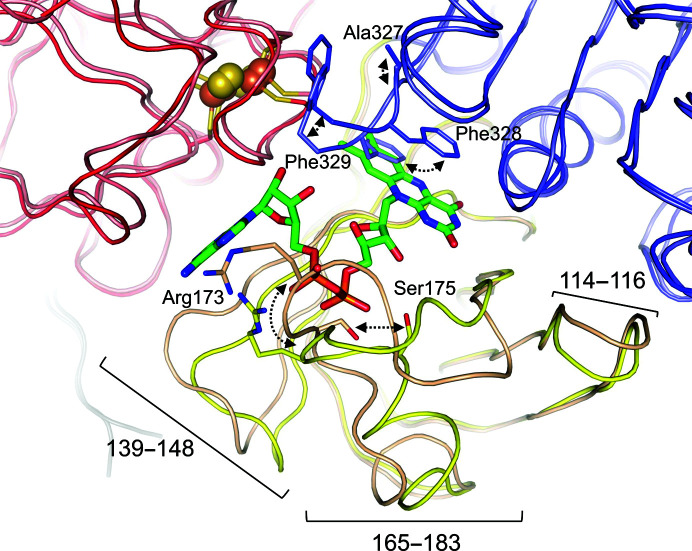
Comparison of structures with and without FAD. Two structures are represented and are coloured as in Fig. 1 ▸. The regions with major structural differences revealed through superposition are illustrated. Arg173 and Ser175, which undergo significant structural differences and form hydrogen bonds to FAD with their side chains, are shown. Residues near the C-terminus (Ala327, Phe328 and Phe329) that are markedly shifted are also shown. Black dashed arrows indicate the movements of these amino-acid residues.

On superimposing the type I and type II structures, conformational differences were observed in several regions of the FAD-binding domain (Fig. 3 ▸). Residues 165–183 in the apo type II structure lacking FAD shifted greatly towards the FAD-binding site to fill space, and the α-helix of residues 175–180 was not formed. Two loops (residues 114–116 and 139–148) moved to fit into the shifted region at residues 165–183. Amino-acid residues in these regions formed hydrogen bonds to FAD, which may be important for accurate binding of FAD. Interestingly, a significant difference was found in the locations of three C-terminal amino-acid residues (Ala327, Phe328 and Phe329) between the two structures (Fig. 3 ▸). In particular, movement of the side chain of Phe328 was required to make space for the isoalloxazine ring of FAD, suggesting high flexibility in this region. For further structural comparison we used the type I structure of CARDO-R_J3_, as the FAD-bound form exhibits activity as an electron-carrier protein.

### 3.3. Ferredoxin (Fd) domain   

**Figure 4:**
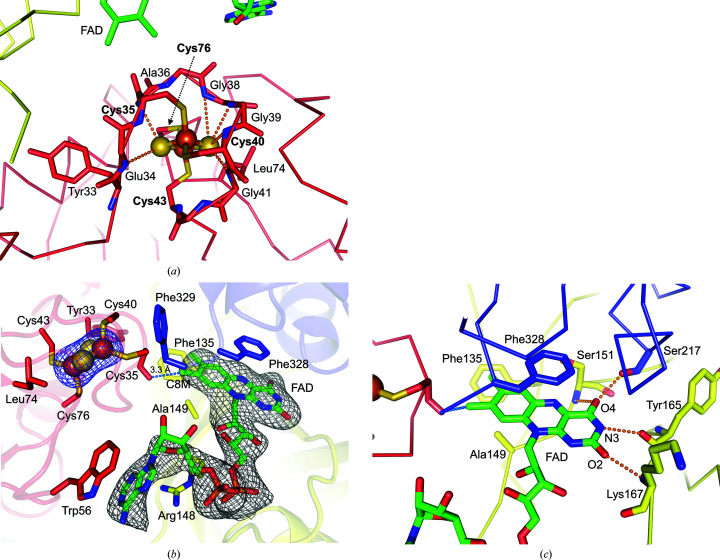
Environment surrounding the redox centres in CARDO-R_J3_. (*a*) The network of hydrogen bonds around the [2Fe–2S] cluster. The individual domains and the [2Fe–2S] cluster are represented and coloured as in Fig. 1 ▸. The [2Fe–2S] cluster is coordinated by cysteine residues 35, 40, 43 and 76 (labelled in bold). The side chains of Tyr33 and Leu74 create a hydrophobic environment within the cluster. Hydrogen bonds between the cluster sulfides and the surrounding residues are shown as orange dashed lines. (*b*) Relationships between redox centres. The difference density maps (*F*_o_ − *F*_c_) of the [2Fe–2S] cluster and FAD in CARDO-R_J3_ are displayed. Both cofactors were omitted for the calculation of the difference density maps, which are shown in blue and grey and contoured at 5.0σ and 2.5σ, respectively. Cys35 O provides the closest approach to C8M of FAD, with an average distance of 3.3 Å. Phe135 in the FAD-binding domain and Phe328 in the NADH-binding domain provide π-stacking interactions and Ala149 supports hydrophobic interaction with the FAD isoalloxazine ring. Arg148 in the FAD-binding domain and Trp56 in the Fd domain interact with adenine and the FAD-binding region via cation–π and π-stacking interactions, respectively. Phe329, which interacts with the Fd domain, is the C-terminal residue of the enzyme. (*c*) Hydrogen bonds between the FAD isoalloxazine ring and the surrounding residues (Ser151, Tyr165, Lys167 and Ser217) are shown.

In CARDO-R_J3_, the Fd domain consists of an acutely twisted β-sheet and three short α-helices. The overall folding of the Fd domain showed high similarity to plant-type ferredoxins (Fukuyama, 2004 ▸). The Fd domain contains a [2Fe–2S] cluster coordinated by four cysteine residues in the Cys-*X*_4_-Cys-*X*_2_-Cys-*X_n_*-Cys motif (Rypniewski *et al.*, 1991 ▸). In CARDO-R_J3_, Cys35, Cys40, Cys43 and Cys76 coordinate the Fe atoms of the [2Fe–2S] cluster, while the main-chain N atoms of residues 34, 36 and 38–41 form hydrogen bonds to the S atoms in the cluster (Figs. 4 ▸ *a* and 4 ▸ *b*). In addition, the cluster is surrounded by the side chains of Tyr33 and Leu74, which create a hydrophobic environment (Fig. 4 ▸ *a*).

In the CARDO-R_J3_ structure, the position of the main-chain O atom of Cys35 (Cys35 O) was closest to the methyl group at the C8 position (C8M) of FAD (average distance of 3.3 Å), suggesting that this orientation would be preferable for electron transfer between the two redox centres. The assumption that these two atoms are likely to take part in the electron-transfer reaction is supported by the results obtained from calculations using *HARLEM*, a program for predicting electron-transfer pathways (Kurnikov, 2003 ▸). However, we must consider the possibility that this orientation may be altered by the binding of NAD(P)H.

On the other hand, comparison of the structural configuration of Cys40 O in CARDO-R_J3_ with those of other plant-type ferredoxins indicated that the orientation of Cys40 O is most similar to that associated with the [2Fe–2S] cluster in the one-electron-reduced state, as observed in putidaredoxin (Sevrioukova, 2005 ▸) and T4moF (Acheson *et al.*, 2015 ▸) (Supplementary Fig. S3). It is possible that the cluster may be reduced by photoreduction from synchrotron radiation because Cys40 O faces ‘out’ even though the crystals of CARDO-R_J3_ were prepared under aerobic conditions.

### 3.4. FAD-binding domain   

The FAD-binding domain of CARDO-R_J3_ is similar to those of BZDO-R (Karlsson *et al.*, 2002 ▸) and T4moF (Acheson *et al.*, 2015 ▸), which are smaller than the corresponding domains in other members of the FNR-like superfamily (Bruns & Karplus, 1995 ▸). The domain is mainly made up of a six-stranded β-sheet and an α-helix, which form a cleft into which the isoalloxazine and ribityl moieties of FAD can bind (Figs. 1 ▸ *a* and 2 ▸).

The relationship between FAD and the [2Fe–2S] cluster in the Fd domain, including important coordinated residues around the redox centre, is shown in Fig. 4 ▸(*b*). Residues in the FAD-binding domain (Ser151, Tyr165, Lys167 and Ser217) form hydrogen bonds to the FAD isoalloxazine ring (Fig. 4 ▸ *c*). Phe135 in the FAD-binding domain and Phe328 in the NADH-binding domain exhibit π-stacking interactions, and hydrophobic interactions with Ala149 stabilize the FAD isoalloxazine ring, sandwiching it from the top and bottom (Figs. 4 ▸ *b* and 4 ▸ *c*). Therefore, the average *B* factors of the FAD isoalloxazine ring (53 Å^2^) were lower than those of the rest of the FAD molecule (70 Å^2^). The C-terminus of the NADH-binding domain is involved in FAD binding in most FNR-like proteins. Phe328 is homologous to Phe325 in T4moF, Phe335 in BZDO-R, Phe225 in PDO-R and Tyr314 in maize leaf FNR–Fd (Fig. 2 ▸), all of which exhibit a π-stacking interaction with an aromatic residue on the isoalloxazine ring of flavin (Figs. 4 ▸ *b* and 4 ▸ *c*).

Two important interactions occur between the protein and the adenine moiety of FAD in the FAD-binding region: a cation–π interaction (Arg148 of the FAD-binding domain) and a π-stacking interaction (Trp56 of the Fd domain), as observed in T4moF (Fig. 4 ▸ *b*). This contribution of the Fd domain is unique compared with observations on other FNRs [maize leaf FNR–Fd (PDB entry 1gaq), *Anabaena* FNR–Fd (PDB entry 1ewy) and BZDO-R (PDB entry 1krh)], where the adenine moiety-stabilizing interactions are supported either by the FAD-binding domain alone or by cooperation between the FAD- and NADH-binding domains (Kurisu *et al.*, 2001 ▸; Morales *et al.*, 2000 ▸; Karlsson *et al.*, 2002 ▸). Although the CARDO-R_J3_ and BZDO-R components are both involved in RO, a notable difference exists in the role of the Fd domains in FAD binding, which might be caused by their distinctive electron-transfer partners: the Fd component for CARDO-R_J3_ and the oxygenase component for BZDO-R. However, the average *B* factors for the adenine moiety of FAD (81 Å^2^) were higher than those for the rest of FAD (60 Å^2^), suggesting that the adenine moiety was not tightly bound.

### 3.5. NADH-binding domain   

The NADH-binding domain consists of a five-stranded β-sheet surrounded by five α-helices, which is typical of the FNR-like superfamily (Ingelman *et al.*, 1997 ▸). In CARDO-R_J3_, π-stacking occurs between the side chain of the penultimate C-terminal residue, Phe328, in the NADH-binding domain and the isoalloxazine ring of FAD, while the C-terminal tyrosine residues are involved in this interaction in photosynthetic FNRs such as maize leaf FNR–Fd (Figs. 2 ▸, 4 ▸ *b* and 4 ▸ *c*; Karplus *et al.*, 1991 ▸; Serre *et al.*, 1996 ▸). In addition, as shown in Fig. 4 ▸(*c*), Ser217 in the NADH-binding domain forms a hydrogen bond to the O4 atom of the isoalloxazine ring on the same side as in PDO-R (Correll *et al.*, 1992 ▸).

Even though extensive attempts were made to determine the structure of CARDO-R_J3_ bound to NADH or NADPH, co-crystallization of CARDO-R_J3_ with either NADH or NADPH failed to produce crystals, and NAD(P)H soaking caused the rapid dissolution of crystals. These results appear to be reasonable, as other researchers have reported that the binding of NAD(P)H promoted conformational rearrangement in this family of reductases (Correll *et al.*, 1993 ▸). In fact, the CARDO-R_J3_ structure suggests that the nicotinamide moiety of NAD(P)H must be replaced with Phe328 from the NADH-binding domain, which interacts with the isoalloxazine ring, for the hydride-transfer reaction to occur (Fig. 4 ▸ *b*).

**Figure 5:**
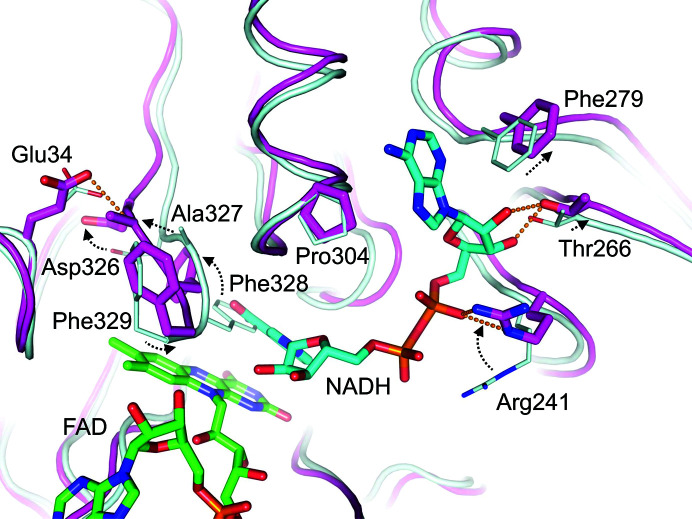
Computer-predicted structure of NADH docking to CARDO-R_J3_. A superimposition of NADH-bound (magenta) and ligand-free (pale cyan) structures is shown. Large shifts when NADH is bound to the binding site are indicated by black dashed arrows. Hydrogen bonds are shown as orange dashed lines.

In previous studies, both NADH and NADPH were effective electron donors for CARDO-R_CA10_ (Nam *et al.*, 2002 ▸). To obtain essential structural insights into the binding of CARDO-R_J3_ by NAD(P)H, pea FNR bound to NADPH (PDB entry 1qfz) was aligned with CARDO-R_J3_ as a template, and NAD(P)H was then incorporated into the modelled CARDO-R_J3_ structure. Additional adjustment of the position of NAD(P)H in CARDO-R_J3_ was carried out considering the interactions around various residues using *Coot* (Emsley *et al.*, 2010 ▸). After building the final models, energy minimization was performed using *Phenix* (Liebschner *et al.*, 2019 ▸; Fig. 5 ▸).

The docking model with NADH revealed that the adenine moiety fits into the cleft between Phe279 and Pro304, while Arg241 coordinates to the pyrophosphate moiety. In addition, Thr266 is likely to form hydrogen bonds to the O2B and O3B atoms of the ribose moiety of NADH. The phenyl ring of Phe328 shifts greatly towards the Fd domain (about 3–5 Å) so that the C4N atom of the nicotinamide moiety is located near the FAD N5 atom to promote the transfer of hydride to FAD. The hydride-transfer reaction is also facilitated by polarization of FAD N1, which is provided by a hydrogen-bonding network involving Ser151, Ser217, Lys167 and the FAD O4 and O2 atoms (Fig. 4 ▸ *c*). Notably, docking of NADH causes a large shift in the positions of the C-terminal residues around Phe328 (Asp326, Ala327 and Phe329), which generates a new hydrogen-bonding inter­action with Glu34 in the Fd domain (Fig. 5 ▸). Incidentally, the NADH-binding domain moves slightly towards the Fd domain. These conformational changes might facilitate electron transfer between the redox centres, FAD and the [2Fe–2S] cluster. In addition, the FAD- and NADH-binding domains appear to move slightly apart in the model with NADH, which has previously been reported in other FNR-type proteins, for example PDO-R (Correll *et al.*, 1992 ▸).

The NADPH-bound model showed similar conformational changes to the NADH-bound model (data not shown). Arg241 forms hydrogen bonds not only to the pyrophos­phate moiety, but also to the phosphate moiety, which contributes to a greatly increased stability of NADPH binding. This result suggests that both NADH and NADPH act as electron donors for CARDO-R_J3_.

### 3.6. Comparison with other reductases   

The structure of CARDO-R_J3_ is the first reported structure of a class III ferredoxin reductase, which can be compared with those of PDO-R (class IA; Correll *et al.*, 1992 ▸) and BZDO-R (class IB; Karlsson *et al.*, 2002 ▸) among the ROs. Through sequence comparison, CARDO-R_J3_, BZDO-R and PDO-R were found to share the same three domains, the Fd, FAD-binding and NAD-binding domains, but these three domains are ordered differently in the proteins (Fig. 2 ▸). The FAD-binding domain is followed by the NADH-binding domain in the amino-acid sequence, but the Fd domain is connected to the C-terminus of the NADH-binding domain in PDO-R and to the N-terminus of the FAD-binding domain in CARDO-R_J3_ and BZDO-R. The structures of PDO-R and CARDO-R_J3_/BZDO-R demonstrate that these three domains are positioned similarly despite their different locations in the sequence. On the other hand, on comparing the sequence and structure of T4moF (Sevrioukova, 2005 ▸; Acheson *et al.*, 2015 ▸), the order and structural positions of the domains are the same in T4moF, CARDO-R_J3_ and BZDO-R.

A detailed comparison of the structures of CARDO-R_J3_ and the oxidoreductases BZDO-R (PDB entry 1krh), PDO-R (PDB entry 2pia) and T4moF (PDB entry 4wqm) was carried out. Superposition of the Fd domains revealed strong agreement, particularly between CARDO-R_J3_ and T4moF, which aligned with an r.m.s.d. of 1.37 Å^2^ (on 97 C^α^ atoms). Likewise, comparison of the FNR-like domains (including both the FAD- and NADH-binding domains) among the four proteins showed r.m.s.d. values of 1.67–2.48 Å^2^. These comparisons indicate that there were minor structural differences among the individual domains.

**Figure 6:**
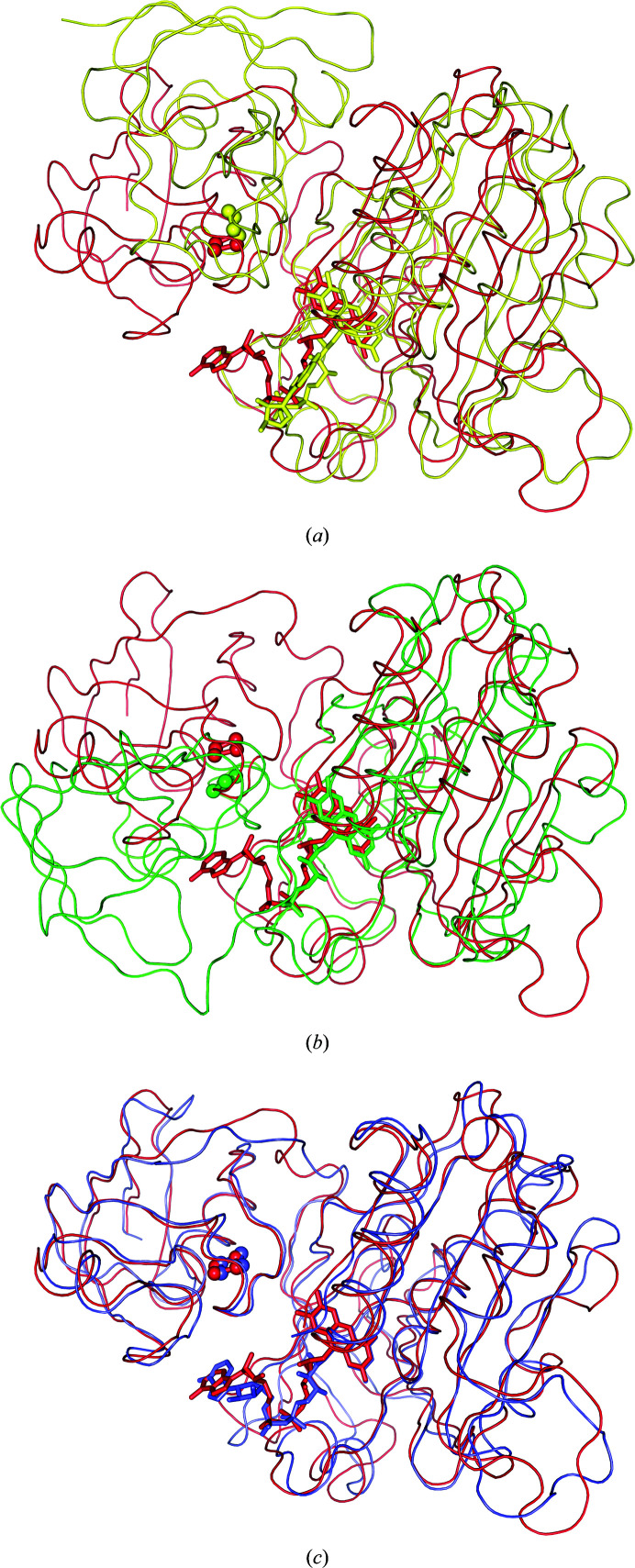
Comparison of the domain arrangement of CARDO-R_J3_ compared with those of other reductases. The FAD- and NADH-binding domains of CARDO-R_J3_ (red) were superimposed on those of (*a*) BZDO-R (yellow; PDB entry 1krh), (*b*) PDO-R (green; PDB entry 2pia) and (*c*) T4moF (slate; PDB entry 4wqm).

**Figure 7:**
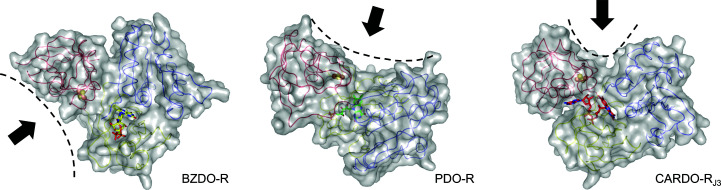
Hypothetical redox-partner-binding regions of RO reductases. Surface plots of reductases are shown (left, BZDO-R, PDB entry 1krh; centre, PDO-R, PDB entry 2pia; right, CARDO-R_J3_). Individual domains and the [2Fe–2S] cluster are represented and coloured as in Fig. 1 ▸ and FAD molecules are coloured as in Fig. 6 ▸. Black dashed lines indicate the surface of the hypothetical binding region of each reductase.

Alignment of the FNR-like domains (the right parts of the molecules shown in Fig. 6 ▸) of CARDO-R_J3_ with BZDO-R (Fig. 6 ▸ *a*), PDO-R (Fig. 6 ▸ *b*) and T4moF (Fig. 6 ▸ *c*) suggested an interesting difference in the relative positions of the Fd domains in their overall structures. The relative positioning of the Fd domains in relation to the other two domains (FNR-like domains) differed significantly, except between CARDO-R_J3_ and T4moF, despite the [2Fe–2S] clusters being located in similar positions. The positions of the [2Fe–2S] clusters in the Fd domains were likely to be constrained by the requirement for efficient electron transfer between the flavin cofactor and the [2Fe–2S] cluster. The distance between Cys35 O, the cysteine residue in the plant-type [2Fe–2S] ferredoxin motif, and the C8M atom of the FAD domain (suggested to be the preferred contact site for electron transfer) in CARDO-R_J3_ was 3.3 Å (Fig. 4 ▸ *b*), which is similar to that in T4moF (3.7 Å). On the other hand, the O atoms of the cysteine residues were located within distances of 5.4 and 4.7 Å in PDO-R and BZDO-R, respectively. Investigation of the crystal-packing patterns of the four protein structures suggested that crystal packing did not affect the positions of the Fd domains (data not shown). In PDO-R and BZDO-R, the positions of the Fd domains were shifted to make space for the possible binding of their redox-partner proteins, *i.e.* the oxygenase components. In PDO-R, this space was a flat surface containing the Fd domain and the NADH-binding domain, while in BZDO-R the position of the Fd domain left space on the other side near the FAD-binding domain (Karlsson *et al.*, 2002 ▸; Fig. 7 ▸). Such differences in the space available for binding may be due to the differing configurations of the oxygenases, which are a homotrimer (α_3_) and a heterotrimer (α_3_β_3_) for the PDO and BZDO systems, respectively. On the other hand, CARDO-R_J3_ and T4moF have similar clefts interposed by Fd and NADH-binding domains, which may fit the arrowhead shape of the relatively small protein ferredoxin (Fig. 7 ▸). These differences are likely to correspond to complementary differences in the structures of their respective redox partners: ferredoxin and terminal oxygenase.

### 3.7. Modelled complex of CARDO-R_J3_ and CARDO-F_CA10_ in the class III CARDO system   

**Figure 8:**
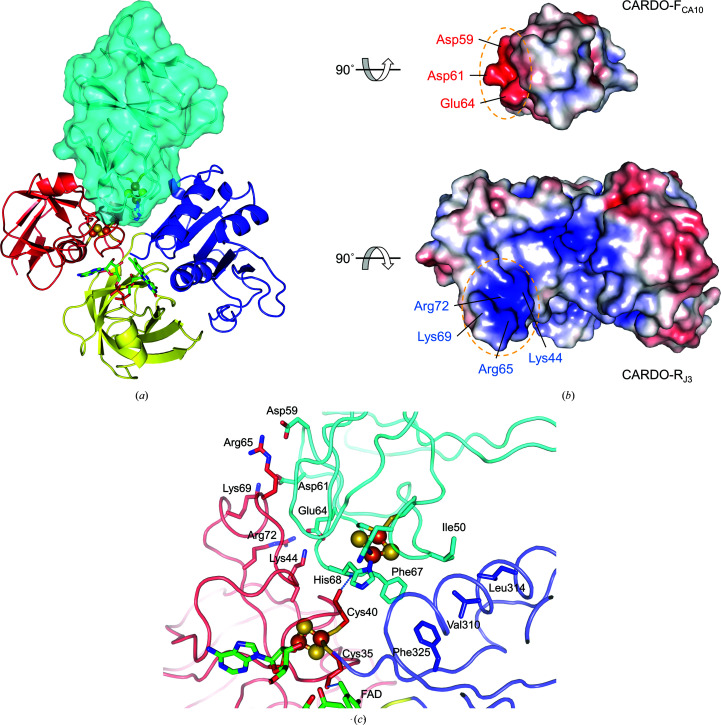
Interactions between CARDO-R_J3_ and CARDO-F_CA10_ in the predicted complex. (*a*) Docking-complex simulation is illustrated with a surface presentation of CARDO-F_CA10_ (cyan). (*b*) Electrostatic surfaces of binding interfaces between CARDO-F_CA10_ (top) and CARDO-R_J3_ (bottom) calculated using *APBS* (Baker *et al.*, 2001 ▸) are shown from −5*kT*/e (red) to +5*kT*/e (blue), with each molecule rotated by 90° to orient the interaction region to the front. The orange dashed circle indicates the interacting regions within the complex. (*c*) Enlarged view of the interface showing key electrostatic and hydrophobic interactions in the predicted complex of CARDO-R_J3_ and CARDO-F_CA10_. The presumptive electron-transport pathway from FAD in CARDO-R_J3_ to the Rieske-type [2Fe–2S] cluster in CARDO-F_CA10_ via Cys40 O and His68 N is shown with teal dashed lines.

We assessed the binding position of the electron-transfer partner CARDO-F_CA10_ on the structure obtained for CARDO-R_J3_ using the protein–protein docking simulation software *ClusPro* (Comeau *et al.*, 2004*a* ▸,*b* ▸). Docking simulations provided several plausible structures for the CARDO-R–CARDO-F complex among a large number of solutions. One of these plausible structures is shown in Fig. 8 ▸(*a*). CARDO-F_CA10_ was bound to the cleft between the Fd and NADH-binding domains of CARDO-R_J3_, resulting in remarkably good steric and electrostatic matching, as described below. Notably, the docked structure placed the [2Fe–2S] clusters of CARDO-F_CA10_ and CARDO-R_J3_ ∼11.0 Å from each other, which is within the predicted suitable distance range for biological electron transfer (Page *et al.*, 1999 ▸). This finding suggests that electron transfer between these redox partners is possible under the binding conditions shown in Fig. 8 ▸(*a*). As shown in Figs. 8 ▸(*b*) and 8 ▸(*c*), the minimum-energy solution showed some interesting features. Several electrostatic and hydrophobic interactions were observed in the proposed complex. An examination of the electrostatic surface potentials of CARDO-R_J3_ and CARDO-F_CA10_ revealed that Coulomb attraction between the two proteins is likely to stabilize the complex. The interface between the two proteins is established in a region that is positively charged on the CARDO-R_J3_ side (Lys44, Arg65, Lys69 and Arg72 in the Fd domain) and negatively charged on the CARDO-F_CA10_ side (Asp59, Asp61 and Glu64) (Figs. 8 ▸ *b* and 8 ▸ *c*). Meanwhile, on the side opposite the static interaction described above, hydrophobic interactions were observed between CARDO-R_J3_ (Val310, Leu314 and Phe325 in the NADH-binding domain) and CARDO-F_CA10_ (Ile50 and Phe67) (Fig. 8 ▸ *c*). Small electron-carrier proteins, such as CARDO-F_CA10_, participate in electron shuttling between the donor and acceptor sites through diffusive encounters and the formation of transient protein complexes (Crowley & Ubbink, 2003 ▸; Crowley & Carrondo, 2004 ▸). Without appropriate docking to bring the redox centres closer and the formation of a long-range electron-transfer route, it has been suggested that diffusional encounters between the two electron-transfer partners would dramatically slow complex formation and electron transfer (Page *et al.*, 1999 ▸). In the CARDO-R_J3_–CARDO-F_CA10_ complex, sufficient electrostatic and structural matching between the two partners (Fig. 8 ▸) would allow efficient electron transfer. As noted above, electron transfer between FAD and the [2Fe–2S] cluster may proceed via FAD C8M and Cys35 O, as predicted by *HARLEM* (Kurnikov, 2003 ▸; Fig. 4 ▸ *b*). In the predicted complex, subsequent electron transfer from the [2Fe–2S] cluster of CARDO-R_J3_ to the Rieske-type [2Fe–2S] cluster in CARDO-F_CA10_ was predicted to occur via Cys40 O and His68 N, coordinating the ligands for each cluster (Fig. 8 ▸ *c*). The stable complex formation with sufficient electrostatic and structural matching described above ensures the presumed electron-transfer pathway, which can be assumed to lead to effective electron transfer.

## 4. Conclusions   

This study of CARDO-R_J3_ provides the first reported structure of a reductase from the class III RO family. The structure obtained here reveals differences in domain arrangement among reductases with different redox partners in their enzyme complexes within the RO family. In addition, based on the results of docking simulations with the redox partner, sufficient electrostatic attraction and shape matching of the interacting regions in the complex could enable efficient electron transfer in the class III RO system.

On the other hand, despite low sequence identity and various permutations of the Fd and FNR-like domains, a high degree of structural homology is observed within the iron–sulfur flavoprotein family to which RO-system reductases belong (Karlsson *et al.*, 2002 ▸). Oxidoreductases are generally considered to have optimized interactions with their cognate protein partners resulting from specific electrostatic and steric interactions among the residues within each electron-transport surface. The present study provides a new example of how interactions between proteins can promote complex formation and electron transfer.

## Supplementary Material

PDB reference: ferredoxin reductase from carbazole 1,9a-dioxygenase, type I structure, 7c3a

PDB reference: type II structure, 7c3b

Supplementary Figures. DOI: 10.1107/S2059798321005040/ji5017sup1.pdf

## Figures and Tables

**Table 1:** Crystal parameters, data-collection statistics and crystallographic refinement statistics Values in parentheses are for the outermost shell.

			SeMet type I crystal for Se-SAD	Type I crystal for Fe-MAD		
Data set	Type I crystal	Type II crystal	Se peak	Fe peak	Fe edge	Fe remote
Crystal data						
Wavelength (Å)	1.00000	1.00000	0.97945	1.73974	1.73993	1.69243
Space group	*P*4_2_2_1_2	*P*4_2_2_1_2	*P*4_2_2_1_2	*P*4_2_2_1_2	*P*4_2_2_1_2	*P*4_2_2_1_2
*a*, *b* (Å)	158.7	161.9	159.6	159.3	159.5	159.6
*c* (Å)	81.5	79.6	81.2	80.9	80.9	80.9
Resolution (Å)	44.01–2.60 (2.69–2.60)	44.91–2.40 (2.49–2.40)	50.00–3.50 (3.63–3.50)	50.00–4.10 (4.25–4.10)	50.00–4.30 (4.45–4.30)	50.00–4.30 (4.45–4.30)
No. of reflections						
Observed	188942	272706	387786	235072	205179	208803
Unique	32288 (3208)	41796 (4118)	13776 (1339)	15592 (1575)	13522 (1358)	13716 (1366)
Completeness (%)	99.1 (99.9)	99.7 (97.5)	100.0 (100.0)	100.0 (99.9)	100.0 (100.0)	99.9 (100.0)
Multiplicity	5.9 (6.0)	6.5 (3.9)	28.1 (27.8)	15.1 (14.1)	15.2 (14.2)	15.2 (14.4)
〈*I*/σ(*I*)〉	8.7 (3.8)	11.4 (3.0)	40.8 (9.2)	36.1 (9.3)	28.4 (9.5)	28.4 (7.9)
*R*_merge_ †	0.088 (0.408)	0.072 (0.412)	0.108 (0.360)	0.114 (0.366)	0.133 (0.399)	0.130 (0.391)
Figure of merit			0.35	0.47		
Refinement						
Resolution (Å)	35.67–2.60 (2.67–2.60)	43.07–2.40 (2.46–2.40)				
*R* factor‡	0.249 (0.341)	0.227 (0.322)				
*R*_free_ §	0.292 (0.406)	0.281 (0.350)				
R.m.s. deviations						
Bond lengths (Å)	0.008	0.007				
Bond angles (°)	1.284	1.255				
Average *B* factors (Å^2^)						
Residues	91.5	70.9				
[2Fe–2S] cluster	50.1	43.7				
FAD	63.9	—				
Waters	56.5	45.6				
Other	61.1	46.2				

† *R*_merge_ = \textstyle \sum_{hkl}\sum_{i}|I_{i}(hkl)- \langle I(hkl)\rangle|/\textstyle \sum_{hkl}\sum_{i}I_{i}(hkl), where *I*_*i*_(*hkl*) is the *i*th observation of reflection *hkl* and 〈*I*(*hkl*)〉 is the weighted average intensity for all observations *i* of reflection *hkl*.

‡ *R* is defined as *R* = \textstyle \sum_{hkl}\big ||F_{\rm obs}|-|F_{\rm calc}|\big |/ \textstyle \sum_{hkl}|F_{\rm obs}|.

§ *R*_free_ was calculated using 5% of the unique reflections.
