# Supplementary material for: Crystal structure of the ferredoxin reductase component of carbazole 1,9a-dioxygenase from Janthinobacterium sp. J3
Source: Acta Crystallogr D Struct Biol. 2021 Jun 18;77(Pt 7):921–32. doi: 10.1107/S2059798321005040 (PMC8251347; doi:10.1107/S2059798321005040)
Supplement: Supplementary file 1 [file d-77-00921-sup1.pdf]

Acta Cryst D
STRUCTURAL
BIOLOGY

Volume 77 (2021)

Supporting information for article:

Crystal structure of the ferredoxin reductase component of carbazole 1,9a-dioxygenase from *Janthinobacterium* sp. J3

Yuji Ashikawa, Zui Fujimoto, Kengo Inoue, Hisakazu Yamane and Hideaki Nojiri

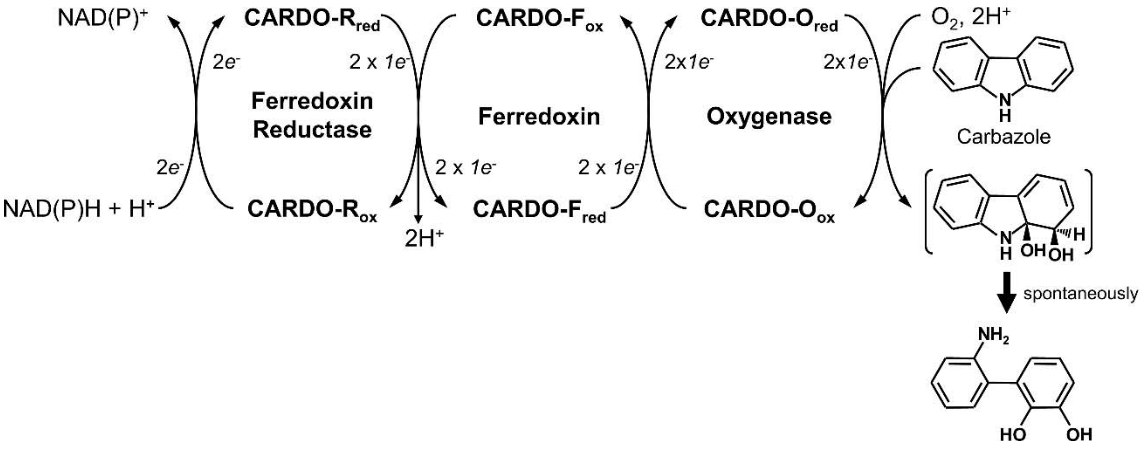

Figure S1 Components and functions of the CARDO system. The proposed electron-transfer reactions and the conversion of carbazole to 2'-aminobiphenyl-2,3-diol are illustrated. The subscripts 'ox' and 'red' indicate oxidized and reduced states of the CARDO components, respectively.

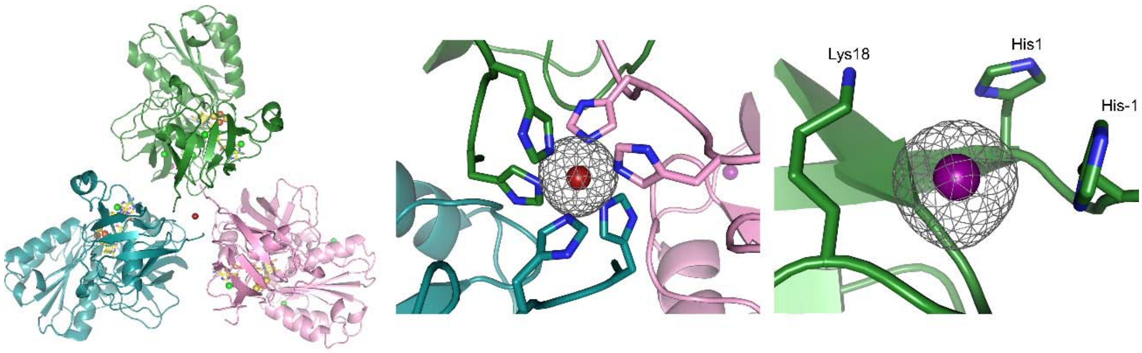

(*a*) (*b*) (*c*)

Figure S2 The type-I CARDO-R_{J3} structure in the asymmetric unit. (a) Three CARDO-R_{J3} molecules were found in the asymmetric unit. Bound FAD (yellow stick) and the [2Fe-2S] cluster (brown and yellow spheres) are shown. Chloride, iodine, and Ni ions are represented as green, purple, and firebrick spheres, respectively. (b) Enlarged view of the site where His-tags at the N-termini of CARDO-R_{J3} molecules are coordinated to Ni ion. The omit map is shown around the Ni ion contoured at 5.0σ. (c) The omit map of an iodine ion surrounding basic residues contoured at 5.0σ.

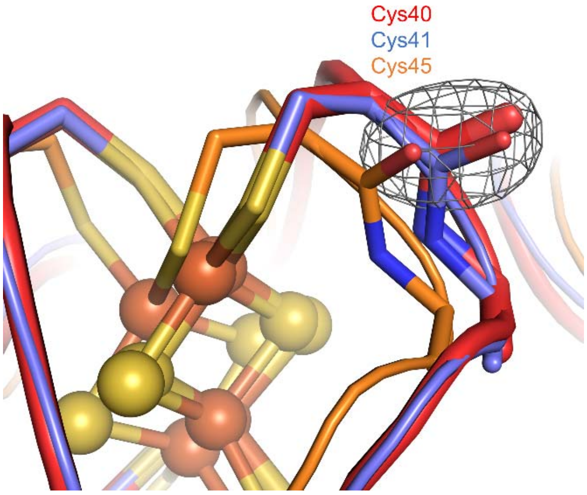

Figure S3 The 'out' conformation of the carbonyl oxygen atom of Cys40 corresponding to its orientation away from the [2Fe-2S] cluster. The Fd domain of CARDO-R_{J3} (red) was superimposed on those of the reduced C73S mutant of Pdx (orange, PDB ID 1xlq) and T4moF (slate, 4wqm), and the omitted density map of the carbonyl group in Cys40 in CARDO-R_{J3} is shown as a grey wire mesh and contoured at 3.5σ.
